# Pathways linking internalized HIV stigma to attitudes and beliefs toward ART through depression: Conditional indirect effects of food insecurity

**DOI:** 10.1371/journal.pone.0350297

**Published:** 2026-06-09

**Authors:** Setor K. Sorkpor, Jerry John Ouner, Rachel G. A. Thompson, Robert Kaba Alhassan, Akua O. Gyamerah, Ibrahim Yigit

**Affiliations:** 1 College of Nursing, Florida State University, Tallahassee, Florida, United States of America; 2 Brain Science and Symptom Management Center, Florida State University, Tallahassee, Florida, United States of America; 3 Department of Family Health Care Nursing, School of Nursing, University of California, San Francisco, California, United States of America; 4 Institute for Global Health Sciences, University of California, San Francisco, California, United States of America; 5 Language Centre, College of Humanities, University of Ghana, Legon, Accra, Ghana; 6 Africa Interdisciplinary Research Institute, Accra, Ghana; 7 School of Health Sciences, University of Dundee, Scotland, United Kingdom; 8 Department of Community Health and Health Behavior, University at Buffalo, New York, United States of America; National Institute of Tuberculosis and Respiratory Diseases, INDIA

## Abstract

**Background:**

Antiretroviral therapy (ART) adherence remains suboptimal in sub-Saharan Africa despite expanded access, particularly in Ghana, where structural and psychosocial stressors such as internalized HIV stigma, depression, and food insecurity interfere with sustained ART engagement. Although these factors are often studied separately, limited research has examined how they interact to influence beliefs and attitudes toward ART.

**Methods:**

We conducted a cross-sectional analysis among 170 adults living with HIV in the Volta Region of Ghana. Participants completed validated measures assessing internalized HIV stigma, depressive symptoms, household food insecurity, beliefs, and attitudes towards ART. We applied mediation and moderated mediation models to test whether depression mediated the association between internalized HIV stigma and beliefs and attitudes toward ART, and whether these indirect effects varied based on food insecurity.

**Results:**

Internalized HIV stigma was associated with more negative beliefs and attitudes toward ART (B = .16, SE = .02, p < .001; B = .08, SE = .02, p < .001). Depression significantly mediated these associations (B = .10, 95% CI [.06, .14]; B = .09, 95% CI [.05, .14]), and food insecurity moderated the association between internalized HIV stigma and depression (B = .36, SE = .13, p = .004). Conditional indirect effects were stronger at high (B = .11, SE = .03, 95% CI [.06, .17]) versus low (B = .06, SE = .02, 95% CI [.03, .10]) food insecurity.

**Conclusions:**

These findings indicate a syndemic interaction between internalized HIV stigma, depression, and food insecurity. Addressing psychological distress alone may not improve ART adherence unless accompanied by efforts to reduce internalized stigma and improve food security. Integrating depression management, stigma reduction interventions, and food security support within HIV services may better enhance treatment engagement in Ghana and similar settings.

## Introduction

Despite considerable global investment in human immunodeficiency virus (HIV) prevention and treatment, achieving optimal adherence to antiretroviral therapy (ART) remains a significant challenge, especially in sub-Saharan Africa (SSA), where approximately two-thirds of all people living with HIV (PLWH) reside [1]. Recent evidence suggests wide variability in ART adherence across the region, with systematic reviews reporting that only 43% to 84% of PLWH achieve optimal adherence [2]. In Ghana, where approximately 350,000 individuals are living with HIV [3], ART uptake has increased, but sustained adherence and viral suppression remain suboptimal due to intersecting structural and psychosocial barriers [3,4]. Thus, understanding the factors that contribute to suboptimal adherence and its underlying causes is crucial for developing tailored interventions to improve the physical and psychological health of PLWH.

Among these barriers, internalized HIV stigma, depressive symptoms, and household food insecurity may be critical determinants of HIV-related health behaviors [5–7]. Depression is a well-established barrier to ART adherence, contributing to diminished motivation, lower self-efficacy, and disengagement from care [5,8]. In Ghana and other low- and middle-income countries (LMICs), depression frequently co-occurs with food insecurity and stigma, stressors that independently predict poor ART outcomes [7,9]. In particular, internalized HIV stigma, defined as the internalization of society’s negative evaluations and perceptions about having HIV and applying them to onesef [6,10] can erode psychological resilience and negatively influence health behaviors through impaired self-worth and heightened distress [6,11,12]. Likewise, food insecurity exacerbates stress and depressive symptoms, further compromising ART engagement [7]. Ghana adopted the World Health Organization (WHO) “Treat All” strategy in 2016 to promote universal ART initiation regardless of CD4 count [13]. While this policy has expanded access, barriers such as long travel distances, medication stockouts, and under-resourced clinics persist. These challenges are particularly acute in the Volta Region, where stigma remains prevalent due to entrenched misinformation, cultural norms, and gaps in mental health services [5,11]. Such structural and social conditions are likely to increase psychological vulnerability and undermine sustained engagement in HIV care.

Although prior research has established that different dimensions of HIV stigma, depression, and food insecurity influence HIV-related outcomes, these factors are often examined in isolation [6,7]. In response, a growing body of literature calls for investigating the mechanisms through which upstream determinants affect health behavior, particularly through pathways involving key psychosocial stressors. Foundational conceptual frameworks, such as those developed by Turan et al. [6] and Weiser et al. [7] emphasize the importance of modeling psychosocial pathways. Recent empirical studies in Ghana build on this conceptual foundation and highlight the interconnected effects of HIV stigma, depression, and food insecurity on HIV-related outcomes [9,11,14]. To advance this line of inquiry, mediation and moderation frameworks allow researchers to disentangle both direct and indirect pathways, offering a more nuanced understanding of these complex psychosocial processes [15]. Prior work among PLWH in the Volta Region of Ghana has shown that internalized stigma and food insecurity are associated with elevated depressive symptoms and ART nonadherence [9,11]. However, the combined effects of these factors and the possibility that depression mediates these associations have not been fully tested.

Beliefs and attitudes toward ART are well-established proximal predictors of treatment adherence. Negative cognitive appraisals, such as fatalism, mistrust in ART efficacy, and low perceived benefit, are consistently associated with delayed initiation and suboptimal retention in care [16]. Affective dimensions, including ambivalence and stigma-related shame, further undermine engagement in HIV treatment [8,17]. Depression may exacerbate these maladaptive cognitive-affective processes, diminishing self-efficacy and motivation to remain in care. Recent evidence suggests that depressive symptoms are significantly associated with lower odds of viral suppression, highlighting the need to address mental health alongside ART adherence [18]. These interrelated psychosocial mechanisms align well with Social Cognitive Theory**,** which posits that a dynamic interaction between personal factors, environmental influences, and behavioral capabilities influences individual behavior [19]. In this context, stigma and food insecurity may affect beliefs and attitudes toward ART through their impact on mental health.

To address this gap, the present study applied a moderated mediation framework to ascertain whether depressive symptoms mediate the relationship between internalized HIV stigma and beliefs and attitudes toward ART, which are established predictors of treatment adherence, and whether these indirect effects are moderated by household food insecurity. Findings will contribute to the understanding of multilevel psychosocial mechanisms influencing ART engagement and inform the design of culturally tailored, integrated interventions in resource-constrained settings.

## Methods

### Data, participants, and procedures

Participants were recruited from a selected HIV clinic in the Volta Region of Ghana during routine clinic visits. A trained clinic nurse identified potentially eligible individuals and introduced the study. Interested patients were referred to research assistants who provided detailed information about the study’s purpose, procedures, potential risks, and confidentiality protections in English or Ewe (predominant local language spoken in the Volta region of Ghana), based on participant preference. Written informed consent was obtained from all participants. Research assistants ensured that participants with limited literacy received full verbal explanations before signing. Participants were reminded that participation was voluntary, and refusal would not affect their access to clinical care. Eligible participants were aged 18 years or older, had an HIV diagnosis for at least six months, and were currently receiving HIV care at the clinic. Exclusion criteria included hospitalization during data collection or a diagnosed severe mental illness that could interfere with participation. Participants received compensation equivalent to $10 USD for their time and transportation costs. Data collection occurred from November 3, 2021 to March 1, 2022.

### Ethical considerations

Ethical approval was obtained from the University of Health and Allied Sciences Research Ethical Committee (UHAS-RECA.6 [1] 20–21) and the University of California, San Francisco Institutional Review Board (20–32955). Permission was granted by Ho Teaching Hospital and the HIV Clinic. We explained the study purpose, procedures, risks, benefits, and confidentiality in the participant’s preferred language. Participants who could not read were provided with an oral explanation of the information sheet. Participation was voluntary, and refusal had no impact on access to care. All eligible individuals approached consented to participate. After signing written informed consent, participants completed the survey and received compensation equivalent to USD 10 for time and travel.

### Measures

#### Internalized stigma.

Internalized stigma was measured using the Internalized Stigma of AIDS Tool [20] consisting of 10 items rated on a 5-point Likert scale (strongly disagree to strongly agree), asking participants to reflect on their thoughts and feelings about themselves since being diagnosed with HIV. Sample items include, “Having HIV infection is like being branded with shame” and “I feel that I need to hide my illness.” A total score was calculated, ranging from 10 to 50, with higher scores indicating higher internalized HIV stigma. The scale demonstrated good internal reliability in our sample, with a Cronbach’s alpha of .86.

#### Household food insecurity.

Household food insecurity was assessed using the Household Food Insecurity Access Scale (HFIAS) [21] consisting of 9 items designed to measure household-level food insecurity related to access over the previous 30 days. Each item assesses whether a specific condition associated with food insecurity occurred (yes/no), followed by how often the condition occurred: rarely (once or twice), sometimes (three to ten times), or often (more than ten times). Responses are scored to produce a total food insecurity score ranging from 0 to 27, with higher scores indicating greater severity of food insecurity. We dichotomized it as 0 (food secure or mildly insecure [low food insecurity]) and 1 (moderately or severely insecure [high food insecurity]). In the current study, Cronbach’s alpha was .93, suggesting excellent internal consistency.

#### Depression.

Depressive symptoms were assessed using the Center for Epidemiologic Studies Depression Scale (CES-D) [22]. The CES-D consists of 20 items rated on a 4-point Likert scale ranging from 0 (rarely or none of the time) to 3 (most or all of the time). Sample items are “I was bothered by things that usually don’t bother me” and “I had trouble keeping my mind on what I was doing.” A total score, ranging from 0 to 60, was calculated, with higher scores indicating greater depressive symptom severity. In the current study, Cronbach’s alpha was .87.

#### Beliefs and attitudes toward ART.

Beliefs about taking ART were assessed using five items adapted from the Theory of Planned Behavior (TPB) [23]. Items were rated on a 5‑point Likert scale (1 = “strongly disagree” to 5 = “strongly agree”). Sample items include “I am confident that I will take my ART drugs consistently as prescribed by my health care provider” and “If I take my ART drugs and experience side effects, I will report them to my healthcare provider.” Total scores (range: 5–25) were reverse‑scored to reflect more negative beliefs.

Attitudes toward ART were measured using 8 items adapted from the TPB [23]. Items were rated on a 5‑point Likert scale (1 = “strongly disagree” to 5 = “strongly agree”) and included statements such as “Taking ART drugs is easy for me” and “I feel comfortable about talking to my healthcare provider about taking my ART drugs.” Total scores (range: 8–40) were reverse-scored to indicate more negative attitudes.

### Data analysis

Descriptive statistics were calculated for all study variables. Next, simple mediation analyses were conducted to examine whether internalized HIV stigma was associated with negative beliefs or negative attitudes toward ART through depression. Additionally, moderated mediation models were tested to assess whether food insecurity status (low vs. high) moderated the indirect effect of internalized HIV stigma on negative beliefs or negative attitudes toward ART through depression, that is, to examine conditional indirect effects at different levels of food insecurity. Hayes’s PROCESS macro (Model 4 for simple mediation models and Model 7 for moderated mediation models) was used, with 95% percentile confidence intervals (CIs) based on 2,000 bootstrapping resamples [24]. According to this procedure, CIs that do not include zero indicate significant indirect effects. For significant interaction (moderation) effects, simple slopes were examined at different levels of food insecurity (low vs. high). The index of moderated mediation was also computed to assess the significance of conditional indirect effects (moderated mediation pathways). All analyses were cross-sectional and adjusted for age, education, and marital status. Unstandardized coefficients were reported using mean-centered continuous variables.

## Results

### Descriptive statistics

Descriptive statistics are presented in Table 1. The final analytic sample included 170 participants. The mean age was 46.61 years (SD = 12.57), with a range of 18–85 years. The majority identified as male (59.4%), 14.7% identified as female, and 25.9% did not report their gender. Slightly more than half of participants were in a relationship (55%), and 45% were single or widowed. Participants were nearly evenly split between urban (50.6%) and rural (49.4%) residences. In terms of education, nearly half (48.5%) had completed primary education. Most participants reported a monthly income of ≤1000 Ghanaian Cedi (GH₵) (85.2%). Regarding food insecurity, 38.0% of participants experienced high levels of household food insecurity, while nearly 62.0% reported low food insecurity. Mean scores were 26.38 (SD = 8.55) for internalized HIV stigma, 9.11 (SD = 8.80) for depression, 23.01 (SD = 2.87) for negative beliefs toward ART, and 27.05 (SD = 2.85) for negative attitudes toward ART.

**Table 1:** Descriptive Statistics (N = 170).

Variables	n	%
Gender		
Female	25	14.7
Male	101	59.4
Missing	44	25.9
Relationship Status		
Single/Widowed	76	45.0
In Relationship	93	55.0
Residence		
Rural	84	49.4
Urban	86	50.6
Education		
None	18	10.7
Primary	82	48.5
Secondary	48	28.4
Post-secondary	21	12.4
Monthly Income		
≤1000 GH₵	132	85.2
>1000 GH₵	23	14.8
Household Food Insecurity		
Low	105	61.8
High	65	38.2
**Variables (*continuous*)**	**M [range]**	**SD**
Age, years	46.61 [18-85]	12.57
Internalized HIV stigma	26.38 [10-45]	8.55
Depression	9.11 [0-35]	8.80
Beliefs toward ART	23.01 [14-25]	2.87
Attitudes toward ART	27.05 [18-30]	2.85

### Mediation analyses

**Fig 1:**
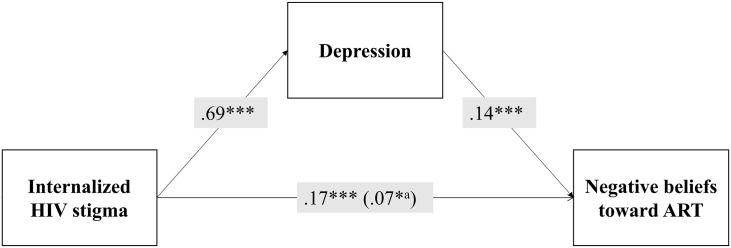
Indirect Effect of Internalized HIV stigma on Negative Beliefs toward ART through Depression. *p < .05, ***p < .001. ^a^The effect when the mediator [depression] is in the model (i.e., direct effect).

**Fig 2:**
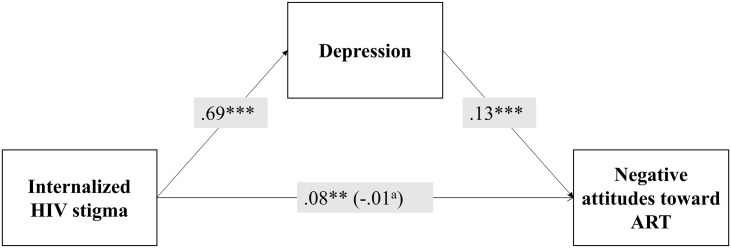
Indirect Effect of Internalized HIV stigma on Negative Attitudes toward ART through Depression. **p < .01, ***p < .001. ^a^The effect when the mediator [depression] is in the model (i.e., direct effect).

We tested whether depression mediated the relationship between internalized HIV stigma and negative beliefs or negative attitudes toward ART (see Figs 1 and 2). Total effect of internalized HIV stigma on negative beliefs toward ART (the effect when the mediator is not in the model) was significant (B = .16, SE = .02, p < .001). The indirect effect was also significant (B = .10, SE = .02, 95% CI [.055, .140]), suggesting that internalized HIV stigma was associated with greater depression (B = .69, SE = .06, p < .001), which in turn was associated with negative beliefs toward ART (B = .14, SE = .03, p < .001). Similarly, the total effect of internalized HIV stigma on negative attitudes toward ART was significant (B = .08, SE = .02, p < .001). The indirect effect was also significant (B = .09, SE = .02, 95% CI [.046, .141]), suggesting that internalized HIV stigma was associated with higher levels of depression (B = .69, B = .06, p < .001), which in turn was associated with negative beliefs toward ART (B = .13, SE = .03, p < .001).

### Moderated mediation analyses

**Fig 3:**
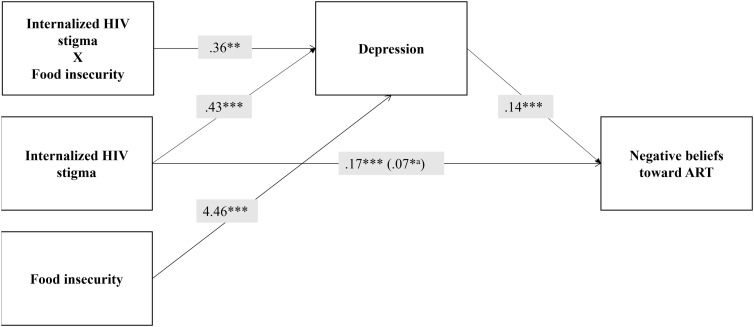
Conditional Indirect Effect of Internalized HIV Stigma on Negative Beliefs toward ART. *p < .05, **p < .01, ***p < .001. ^a^The effect when the mediator [depression] is in the model (i.e., direct effect).

**Fig 4:**
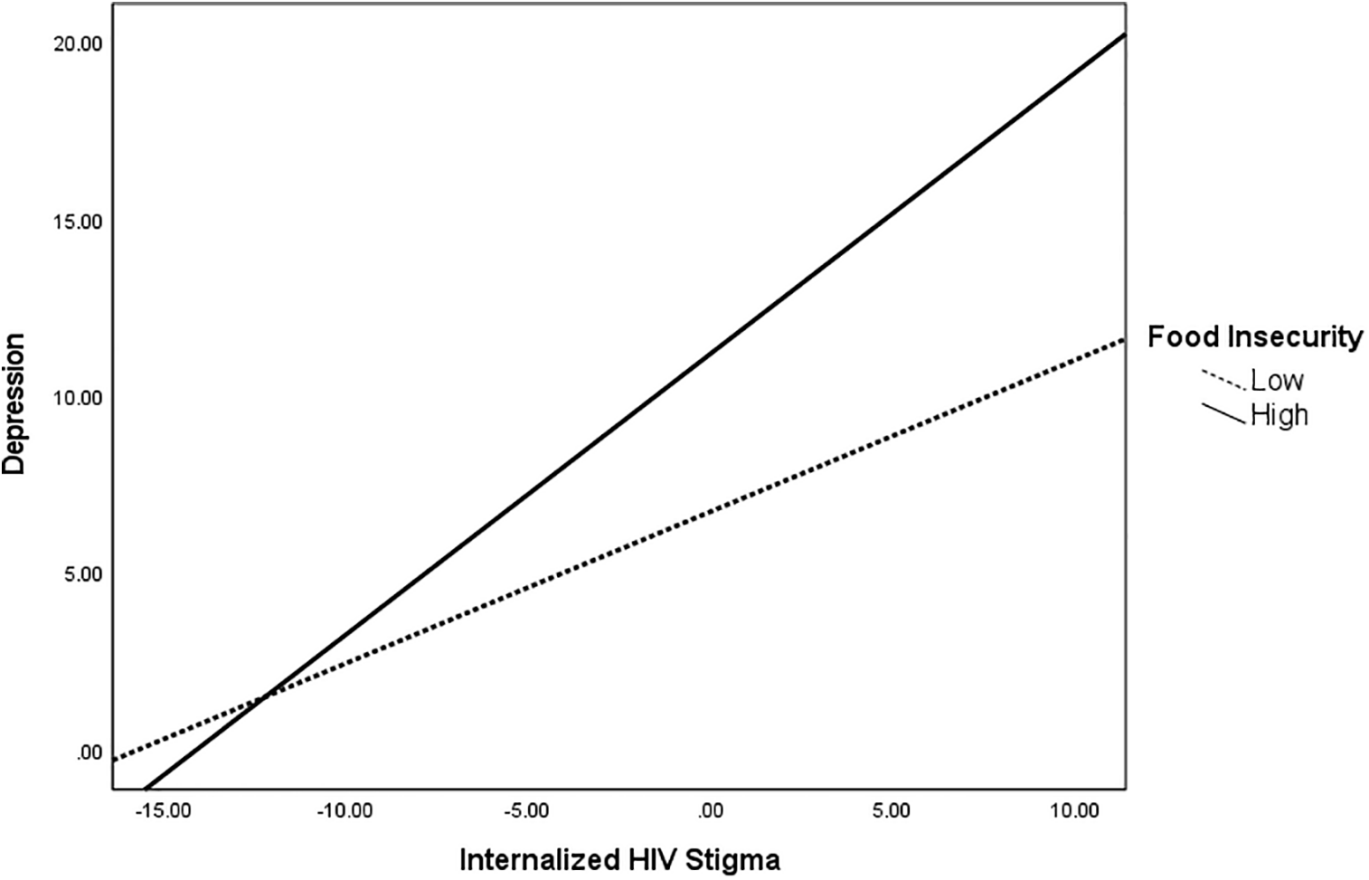
Simple Slope Analysis for the Interaction between Internalized HIV Stigma and Food Insecurity on Depression.

We conducted moderated mediation analyses to examine the conditional indirect effects of internalized HIV stigma on negative beliefs or negative attitudes toward ART through depression at different levels of food insecurity. In the first model (see Fig 3), with negative beliefs toward ART as the outcome, food insecurity was significantly associated with depression (B = 4.46, SE = 1.10, p < .001). The interaction effect between internalized HIV stigma and food insecurity on depression was significant (B = .36, SE = .13, p = .004). As shown in the simple slope analysis (see Fig 4), internalized HIV stigma was more significantly associated with depression at high levels of food insecurity (B = .79, SE = .10, 95% CI [.598, .990]) compared to low levels of food insecurity (B = .43, SE = .08, 95% CI [.274, .583]). Furthermore, the conditional indirect effect was also significant (index of moderated mediation: B = .05, SE = .02, 95% CI [.017, .095]). This result indicates that the indirect effect of internalized HIV stigma on negative beliefs toward ART through depression was stronger at high levels of food insecurity (B = .11, SE = .03, 95% CI [.062, .167]) compared to low levels of food insecurity (B = .06, SE = .02, 95% CI [.030, .095]).

**Fig 5:**
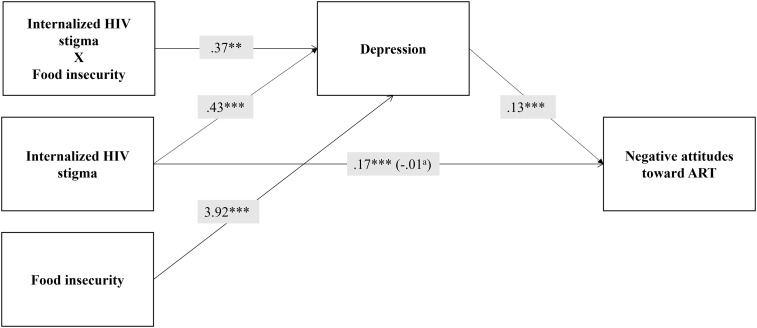
Conditional Indirect Effect of Internalized HIV Stigma on Negative Attitudes toward ART. **p < .01, ***p < .001. ^a^The effect when the mediator [depression] is in the model (i.e., direct effect).

Next, we tested the second moderated mediation model with negative attitudes toward ART (see Fig 5). As in the first moderated mediation model, the effect of food insecurity on depression was significant (B = 4.46, SE = 1.10, p < .001). Similarly, the interaction effect between internalized HIV stigma and food insecurity on depression was significant (B = .36, SE = .13, p = .004), with a stronger association between internalized HIV stigma and depression at high levels of food insecurity (see Fig 3). The conditional indirect effect was also significant (index of moderated mediation: B = .05, SE = .02, 95% CI[.013, .102]), indicating that the indirect effect of internalized HIV stigma on negative attitudes toward ART through depression was stronger at high levels of food insecurity (B = .10, SE = .03, 95% CI[.050, .174]) compared to low levels of food insecurity B = .06, SE = .02, 95% CI[.028, .092]).

## Discussion

This study examined the pathways linking internalized HIV stigma to beliefs and attitudes toward ART through depression, and whether food insecurity moderated these indirect effects among PLWH in Ghana. Our study applied a novel moderated mediation framework to explore how structural and psychosocial stressors, namely internalized stigma, depression, and food insecurity, interact to influence these cognitive-affective mechanisms, which are established predictors of ART adherence. These findings highlight the importance of addressing both psychological and structural factors that jointly undermine ART adherence.

The findings are consistent with a substantial body of evidence indicating that internalized HIV stigma undermines treatment engagement by eroding psychological resilience and disrupting motivation for care. Internalized stigma has been shown to compromise self-worth and heighten social withdrawal, thereby contributing to depressive symptoms and diminished ART adherence [25,26]. In a seminal multisite study of women living with HIV in the United States, Turan et al. [25] found that the effect of internalized stigma on ART adherence was mediated by both social isolation and depression, affecting emotional and relational functioning in ways that closely resemble those observed in our Ghanaian sample. Similarly, Turan et al. [26] found that depressive symptoms were a central pathway linking stigma to disengagement from care, affirming that cognitive-affective disruptions play a critical role in beliefs and attitudes toward ART. Complementary evidence from Senegal reported by Benzekri et al. [27] extends this conceptual understanding by showing that internalized HIV stigma operates within broader structural vulnerabilities, including poverty, unemployment, and social exclusion, which frequently intersect with food insecurity to undermine engagement in HIV care. These findings reinforce the notion that internalized HIV stigma is not merely an individual-level phenomenon but deeply embedded within broader social and material contexts, consistent with our observed interaction between internalized stigma, depression, and food insecurity.

In Ghana, Ouner et al. [11] also identified a significant negative association between internalized HIV stigma and ART adherence among adults living with HIV; however, depression did not mediate this relationship. This divergence likely reflects differences in analytic strategy, as our moderated mediation analysis was designed to capture conditional indirect effects, revealing how food insecurity amplifies the psychological burden of HIV stigma. This syndemic association between psychosocial vulnerability (internalized stigma and depression) and structural deprivation (food insecurity) underscores the importance of multilevel interventions that address both mental health and economic precarity in efforts to promote sustained ART engagement in resource-limited settings [28]. A more nuanced understanding of these intersecting burdens may enhance targeting intervention and inform policy approaches aimed at optimizing HIV outcomes in high-vulnerability contexts.

These findings provide preliminary insights that may help refine interventions addressing stigma, depression, and structural vulnerabilities such as food insecurity. Addressing stigma and depression in isolation may be insufficient unless accompanied by targeted efforts to alleviate structural vulnerabilities such as food insecurity. Integrated care models that combine psychosocial counseling with food or economic support have demonstrated greater efficacy in promoting sustained ART engagement among PLWH facing intersecting burdens [29]. For example, incorporating routine screening for food insecurity and internalized stigma into HIV care visits could help providers identify at-risk individuals and connect them to holistic support services. Syndemic-aware HIV care systems may offer a more effective pathway to achieving sustained viral suppression, particularly in high-vulnerability settings such as Ghana. Although these findings are preliminary and not yet sufficient to directly inform policy, they provide important insights that warrant future research to develop evidence-based policy interventions addressing both psychological and structural drivers of disengagement in tandem. This aligns with recent research from Ghana demonstrating that food insecurity independently predicts lower ART adherence, highlighting the need to consider material deprivation in adherence-focused interventions [11]. In response, HIV programs must move toward multilevel models that integrate mental health care, stigma reduction, and food security support. Peer-led psychosocial interventions in Ghana, such as the HIV Education, Empathy, and Empowerment (HIVE³) peer support model, have demonstrated preliminary acceptability and potential to reduce intersectional stigma and improve engagement in HIV care [30]. Similarly, integrated models that combine psychosocial counseling with structural supports, including food or economic assistance, have demonstrated feasibility and acceptability in other sub-Saharan African contexts [31–33], and warrant adaptation to Ghana’s healthcare system.

Our findings provide empirical evidence of the cognitive-affective mechanisms through which internalized HIV stigma contributes to treatment disengagement, particularly within contexts marked by material deprivation such as food insecurity. This pathway aligns with cognitive-behavioral theory, which posits that depression contributes to distorted beliefs, diminished perceived benefits of care, and reduced self-efficacy, ultimately undermining treatment motivation [34^,35].^ In high-vulnerability settings, where social marginalization and resource constraints often coexist, these psychological disruptions are further intensified by structural stressors such as food insecurity. The synergistic effect of these psychosocial and structural burdens exemplifies a syndemic process that erodes confidence in one’s ability to adhere to ART [36,37]. By highlighting these intersecting mechanisms, our findings reinforce the need for multilevel interventions that simultaneously address stigma, mental health, and structural deprivation to support sustained engagement in HIV care [6,7].

This study contributes to the growing literature on multilevel influences on ART engagement in resource-limited settings by examining how internalized HIV stigma and food insecurity interact to shape treatment outcomes through depressive symptoms. The use of validated measures for key constructs enhances the credibility of the findings, and the application of bootstrapped moderated mediation models with covariate adjustment provides a rigorous analytic approach for evaluating conditional psychosocial pathways. However, several limitations should be acknowledged. First, the cross-sectional design limits causal inference and precludes temporal conclusions regarding mediation. Second, the reliance on self-reported measures of all study variables may introduce bias due to social desirability or recall inaccuracies. Third, although the study was conducted in a diverse region of Ghana, the findings may not be generalizable to other national or regional contexts. Future research should replicate these findings using longitudinal designs and consider integrating clinical outcomes such as viral load suppression and other biomarkers.

## Conclusion

Our findings suggest that addressing psychological vulnerabilities alone may be insufficient to sustain ART engagement in high-burden settings unless efforts to alleviate structural deprivation are made. The observed conditional indirect effects indicate that food insecurity amplifies the emotional and cognitive toll of internalized HIV stigma, leading to heightened depressive symptoms and shaping more negative beliefs and attitudes toward ART, which are critical to treatment adherence. Integrating mental health services, stigma reduction, and food security interventions into HIV care may be essential to improving outcomes in Ghana. A multilevel, syndemic-aware approach tailored to local needs holds promise for promoting equitable and sustainable engagement in HIV care. Future research using larger, multi-site and multi-region studies are needed to confirm these findings and guide the development of scalable, evidence-based interventions.

## Supporting information

S1 File Supporting Information_ Inclusivity in global research. (DOCX)

S2 File PLOS inclusivity questionnaire for global research. (DOCX)
