# Supplementary material for: Pathways linking internalized HIV stigma to attitudes and beliefs toward ART through depression: Conditional indirect effects of food insecurity
Source: PLoS One. 2026 Jun 9;21(6):e0350297. doi: 10.1371/journal.pone.0350297 (PMC13249159; doi:10.1371/journal.pone.0350297)
Supplement: S2 File — (DOCX) [file pone.0350297.s002.docx]

Inclusivity in global research

PLOS’ policy on inclusivity in global research aims to improve transparency in the reporting of research performed outside of researchers’ own country or community and ensures that PLOS publications reporting global research adhere to high standards for research ethics and authorship. Authors of relevant research articles may be asked to complete the questionnaire below, which outlines ethical, cultural, and scientific considerations specific to inclusivity in global research. This questionnaire may be requested when researchers have travelled to a different country to conduct research, if research uses samples collected in another country, research with Indigenous populations or their lands, or if research is on cultural artefacts. Researchers travelling to another country solely to use laboratory equipment will not normally be required to complete the questionnaire. However, the questionnaire can be requested at the journal’s discretion for any submission – if you have been requested to complete this questionnaire by the PLOS journal you submitted to, please do so.

Please complete the questionnaire below and include this as a Supporting Information file with your manuscript. Note that if your paper is accepted for publication, this checklist will be published with your article in the supporting information files. Please ensure that you reference the checklist in the main body of your manuscript. We suggest adding a subsection ‘Inclusivity in global research’ to your Methods section and adding the following sentence: “Additional information regarding the ethical, cultural, and scientific considerations specific to inclusivity in global research is included in the Supporting Information (SX Checklist)”

The questions have been designed to be applicable to a wide range of study types, and there are subsections for both human subjects research and non-human subjects research. If any of the questions are not relevant to your research please mark them as “N/A” as appropriate.

**Ethical considerations, permits and authorship**

*This section is applicable to all research types.*

Provide details as to who granted permissions and/or consent for the study to take place in the Methods section of your manuscript. This should include the names of **all** ethics boards, governmental organizations, community leaders or other bodies that provided approval for the study. If individuals provided approval refer to these people by their role or title but do not list their name(s).

Reported on page number: 7 Ethical approval was obtained from the University of Health and Allied Sciences Research Ethical Committee (UHAS-RECA.6 [1] 20–21) and the University of California, San Francisco Institutional Review Board (20–32955). Permission was granted by Ho Teaching Hospital and the HIV Clinic. We explained the study purpose, procedures, risks, benefits, and confidentiality in the participant’s preferred language. Participants who could not read were provided with an oral explanation of the information sheet. Participation was voluntary, and refusal had no impact on access to care. All eligible individuals approached consented to participate. After signing written informed consent, participants completed the survey and received compensation equivalent to USD 10 for time and travel. Depressive symptoms were assessed as part of the research survey and were not used for diagnostic or clinical decision-making purposes. Participants were already receiving routine HIV care at the clinic where mental health services are available through standard care pathways.

If there were any deviations from the study protocol after approval was obtained please provide details of these changes in the Methods section of your manuscript.
Did this study involve local collaborators that are residents of the country where the research was conducted or members of the community studied? If you do not have any authors from said communities, please provide an explanation for this below.

Yes. The study involved local collaborators, including Dr. Robert Kaba Alhassan and Dr. Rachel G.A. Thompson, both residents of Ghana. Dr. Alhassan was previously affiliated with the University of Health and Allied Sciences, Ho, in Ghana where the data was collected and is currently on a temporary appointment at the University of Dundee; however, he continues to reside in Ghana for most of the time. Dr. Thompson is a Ghanaian national and a resident of Ghana. She is also a Global Faculty Affiliate of the University of California, San Francisco. Dr. Ouner, a faculty member at the University of California, San Francisco, is a citizen of Ghana and remains actively involved in initiatives in Ghana.

The local collaborators contributed to the study design, recruitment procedure, data collection and analysis and contextual interpretation of the results

All listed authors meet PLOS’ criteria for authorship.

All listed authors meet PLOS’ criteria for authorship.

Reported on page number: No deviations from the approved study protocol occurred after ethical approval was obtained.

Everyone listed as an author should meet PLOS’ criteria for authorship and all individuals who meet these criteria should be included in the author byline, rather than the acknowledgements. For further information please see the journal’s Authorship Policy.

**Human subjects research (e.g. health research, medical research, cross-cultural psychology)**

Did you obtain written informed consent from a representative of the local community or region before the research took place? How did you establish who speaks for the community? Details of written informed consent obtained from study participants should be reported separately in the Methods section of your manuscript.

Written informed consent was obtained from all individual study participants. Because the study was conducted within an established clinical setting (Ho Teaching Hospital HIV Clinic), institutional approval from the hospital administration and clinic leadership served as site-level authorization for study implementation.

How did members of the local community provide input on the aims of the research investigation, its methodology, and its anticipated outcome(s)?

Local collaborators and clinical staff at Ho Teaching Hospital provided input during study development, including review of study procedures, recruitment processes, and cultural appropriateness of survey instruments. Their input informed implementation within the clinic context.

When engaging with the local community, how did you ensure that the informed consent documents and other materials could be understood by local stakeholders?

Study materials were provided in participants’ preferred language. Research assistants explained the study purpose, procedures, risks, and benefits verbally when needed. Participants who could not read were provided with an oral explanation of the information sheet prior to providing written consent.

Will the findings of the research be made available in an understandable format to stakeholders in the community where the study was conducted (e.g. via a presentation, summary report, copies of publications, etc.)? Please provide details of how this will be achieved.

Yes. Findings will be shared with collaborating institutions and clinic leadership at Ho Teaching Hospital. Results will also be disseminated through academic publications and may be shared in summary form with local clinical partners upon request.

**Non-human subjects research using specimens/ animals collected as part of the study, or those housed in archival collections. Examples include archaeology, paleontology, botany and zoology.**

Did the permission you obtained from a local authority to perform the study include an agreement on access to outputs and benefit sharing? This may include procedures to enable fair distribution of the benefits and resources arising from the research performed. Please include any details of Prior Informed Consent and Benefit Sharing Agreements obtained. These may be required by field-specific regulations, for example the Convention on Biological Diversity (CBD) and the associated Nagoya Protocol.

N/A

If the material used in your study was imported, please A) provide the year it was imported and B) indicate whether permits were obtained to import/export the materials used, C) provide details of any permits obtained. If this information is not available, please indicate this.

N/A

If you used archival specimens, please state how the material used in your study was acquired by the institute it is held in and provide details of any permits obtained for the original excavations/ sample collection. If this information is not available, please indicate this.

N/A

How was the potential cultural significance of the materials collected in your study to local communities considered in your research design? Were Indigenous peoples and/or local researchers and institutions involved with archaeological excavations / collection of specimens? If so, please provide a description of their involvement.

N/A

If your manuscript includes photographs of human remains please indicate whether authors obtained permission from descendants or affiliated cultural communities to do so.

N/A
